# A Precise Annotation of Phase-Amplitude Coupling Intensity

**DOI:** 10.1371/journal.pone.0163940

**Published:** 2016-10-04

**Authors:** Ning Cheng, Qun Li, Xiaxia Xu, Tao Zhang

**Affiliations:** 1 College of Life Sciences and Key Laboratory of Bioactive Materials Ministry of Education, Nankai University, Tianjin, PR China; 2 College of Mathematics, Nankai University, 300071, Tianjin, PR China; Georgia State University, UNITED STATES

## Abstract

Neuronal information can be coded in different temporal and spatial scales. Cross-frequency coupling of neuronal oscillations, especially phase-amplitude coupling (PAC), plays a critical functional role in neuronal communication and large scale neuronal encoding. Several approaches have been developed to assess PAC intensity. It is generally agreed that the PAC intensity relates to the uneven distribution of the fast oscillation amplitude conditioned on the slow oscillation phase. However, it is still not clear what the PAC intensity exactly means. In the present study, it was found that there were three types of interferential signals taking part in PAC phenomenon. Based on the classification of interferential signals, the conception of PAC intensity is theoretically annotated as the proportion of slow or fast oscillation that is involved in a related PAC phenomenon. In order to make sure that the annotation is proper to some content, simulation data are constructed and then analyzed by three PAC approaches. These approaches are the mean vector length (MVL), the modulation index (MI), and a new permutation mutual information (PMI) method in which the permutation entropy and the information theory are applied. Results show positive correlations between PAC values derived from all three methods and the suggested intensity. Finally, the amplitude distributions, i.e. the phase-amplitude plots, obtained from different PAC intensities show that the annotation proposed in the study is in line with the previous understandings.

## Introduction

Neural information can be coded in different scales in brain [1, 2]. Continuous electrophysiological signals, recorded at mesoscopic and macroscopic levels, e.g. local field potential (LFP) and electroencephalogram recordings (EEG), show rhythmical characteristics known as neuronal oscillations. Usually, they are divided into five interactive frequency bands named delta (1-4Hz), theta (4-8Hz), alpha (8-12Hz), beta (12-28Hz), and gamma (28Hz-100Hz) oscillations [3–5]. In addition, neural oscillations play a fundamental role in learning and memory [4, 6]. A number of studies point out that phase synchronization of oscillations at an identical rhythm could support neural communication and facilitate neural plasticity [7–9]. Moreover, oscillations at different rhythms are not isolated. They could interact with each other through a communication mechanism called Cross-Frequency Coupling (CFC) [10]. Obviously, CFC plays a critical functional role in neuronal communication and large scale neuronal encoding, involving in both multi-item working memory maintenance and long-term memory retrieval [11, 12]. Two types of CFC, namely Phase-Phase Coupling (PPC) [13] and Phase-Amplitude Coupling (PAC) [14], have been widely investigated. Noticeably, the cross-frequency coupling not only occurs in one brain region but also delivers information across two connected brain areas [5].

Particular attentions are given to the PAC phenomenon, otherwise referred to as nested oscillation, which is the coupling between the phase of slow oscillation and the amplitude of fast oscillation. This is a phenomenon that the energy of the high frequency band will be modulated by the time course of the low frequency band. It is widely reported in hippocampus, basal ganglia and neocortex from rats, mice, monkeys to humans [12, 15, 16]. Moreover, it is well known that the PAC is involved in sensory information detection, attention selection and memory [17, 18]. In rats’ hippocampus, the phase of theta rhythm may modulate the amplitude of gamma rhythm, which is probably a potential mechanism for the organization of serial memories and maintenance of working memories [19]. One of previous studies showed that the amplitude of gamma reached the highest value in different specific phases of theta in the hippocampal sub-regions [4].

So far, there are several approaches by which PAC can be assessed. The methods have been rather heterogeneous for their diverse purposes of application [20, 21]. Comparisons of different PAC methods have been proposed from different perspective in the literature, for the main purpose of detecting PAC intensity better. However, the conception of PAC intensity is ambiguous to some extent in different studies. It suggests that the intensity of PAC can be inferred by visual inspection of the phase-amplitude plot [22], which is a common understanding of PAC intensity. However, the accurate intensity could not be inferred by the phase-amplitude plot. In the present study, we made a great effort to clarify the PAC intensity properly and accurately.

**Fig 1:**
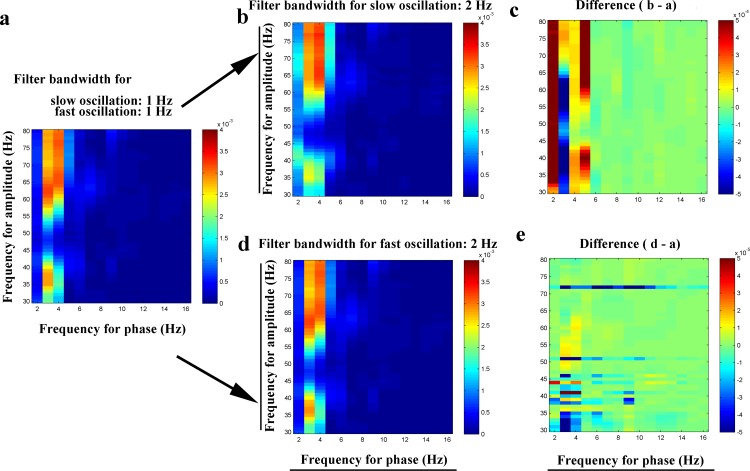
Phase–amplitude coupling between hippocampal DG low frequency rhythm (2–16 Hz, theta and alpha) and high frequency rhythm (30–80 Hz, gamma) measured by Modulation Index. (a) PAC between low frequency rhythm (filter bandwidth = 1 Hz) and high frequency rhythm (filter bandwidth = 1 Hz). (b) PAC between low frequency rhythm (filter bandwidth = 2 Hz) and high frequency rhythm (filter bandwidth = 1 Hz). (c) Difference (b-a). (d) PAC between low frequency rhythm (filter bandwidth = 1 Hz) and high frequency rhythm (filter bandwidth = 2 Hz). (e) Difference (d-a).

First of all, we make a brief classification of variables that may interfere with PAC measurement. As illustrated in the PAC comodulograms from an anaesthetic mouse (Fig 1), it can be seen that there are three types of PAC interference due to the bandwidth filter of the slow oscillation and the fast oscillation. They are: (i) the slow oscillation band covers two different components, one of which modulates the fast oscillation amplitude and not another. For example, if gamma amplitude is modulated by only one theta component, and then the PAC measurement may be interfered with the presence of another theta component. (ii) the fast oscillation band covers two different components, one of which is modulated by the slow oscillation phase and not another. The PAC measurement will be interfered with the presence of the irrelevant oscillation. (iii) the fast oscillation band covers one frequency component, whose amplitude, however, is jointly modulated by two slow oscillations. If the PAC intensity between one of these two slow oscillations and the fast oscillation is assessed by the PAC methods, its measurement will be impacted by the other slow oscillation.

Based on the above classification of variables that may interfere with PAC measurement, for the first time to our knowledge, the conception of PAC intensity is theoretically annotated as the proportion of slow or fast oscillation that is involved in the concerned PAC phenomenon. In the present study, the annotation is utilized throughout all numerical tests. Subsequently, three types of PAC approaches were applied in the numerical test using simulation data to show whether the above annotation of PAC intensity was proper to some extent. These methods are the mean vector length (MVL) [14], the modulation index (MI) [22], and a new permutation mutual information (PMI) method in which the permutation entropy [23] and the information theory are used. Finally, the phase-amplitude plots are utilized to confirm if the annotation is in line with the previous understanding of PAC intensity, i.e. the uneven distribution of the fast oscillation amplitude conditioned on the slow oscillation phase [10, 22].

## Materials and Methods

### Two well-known PAC methods

In this section, two conventional PAC algorithms are introduced, these are, the mean vector length (MVL) and the modulation index (MI). Before introducing the approaches, let *Low*(*t*) and *High*(*t*) denote the slow oscillation and the fast oscillation filtered from raw data, separately.

#### The mean vector length (MVL)

It was proposed by Canolty and his colleagues [14]. The main idea of the method is to create a complex time series (or a vector time series) by multiplying slow oscillation phase series by fast oscillation amplitude. And then the length of the average vector across time is used as the indicator of PAC.

$$MVL=\left|average(A_{High}(t)*e^{i\theta_{Low}(t)})\right|$$ (1)

If the PAC does not exist between the slow oscillation and fast oscillation at all, the amplitude *A*_*High*_(*t*) distribution conditioned on the phase (or the time course) of slow oscillation will be random or uniform, which results to a zero value of MVL. On the contrary, if the amplitude *A*_*High*_(*t*) is modulated by the phase *θ*_*Low*_(*t*), or in other words, the amplitude of fast oscillation reaches the highest value at specific phases of slow oscillation and the lower values at other phases, an obviously positive PMI will be obtained because of the nonuniform distribution of *A*_*High*_(*t*).

In general, a higher MVL value represents a stronger PAC.

#### The modulation index (MI)

The method was proposed by Tort and his colleagues [22], whose primary idea was that there was a nonuniform distribution of amplitude *A*_*High*_(*t*) conditioned on phase *θ*_*Low*_(*t*) when PAC existed between them. The MI method has been developed based on Shannon Entropy of the foregoing distribution and normalized by the largest value of Shannon Entropy when the distribution is uniform.

The detailed procedures of MI are as follows. First of all, both *θ*_*Low*_(*t*) and *A*_*High*_(*t*) are extracted by Hilbert Transform. Afterwards each cycle of *θ*_*Low*_(*t*) is equally divided into *n* intervals. Corresponding to each interval of *θ*_*Low*_(*t*), the average of *A*_*High*_(*t*) can be calculated, which can be normalized by dividing it by the sum of all averages over all intervals to get the distribution of amplitude *A*_*High*_(*t*) conditioned on phase *θ*_*Low*_(*t*).

$$P(j)=\frac{{\left\langle A_{High}(t)\right\rangle }_{\theta }(j)}{\sum_{k=1}^{n}{\left\langle A_{High}(t)\right\rangle }_{\theta }(k)}$$ (2)

And then the Shannon Entropy of the distribution can be quantified:

$$H(P)=-\sum_{j=1}^{n}P(j)*\log[P(j)]$$ (3)

Notice that log(*n*) is the maximal possible value of *H*(*P*), which happens precisely for the uniform distribution when we have *P*(*j*) = 1/*n* for all intervals *j* [22]. Accordingly, *H*(*P*) can be normalized to the so-called Modulation Index (MI),

$$MI=\frac{\log(n)-H(P)}{\log(n)},\;MI\in [0,1]$$ (4)

It is obvious that a larger MI value indicates a more nonuniform distribution of amplitude *A*_*High*_(*t*) conditioned on phase *θ*_*Low*_(*t*), which can be regarded as a stronger intensity of PAC.

### PMI (permutation mutual information)

The details of PMI with relative concepts are as follows. It can be seen that the PAC between two frequency bands can be measured by using the PMI approach. The PMI is developed based on the permutation entropy [23–27] and mutual information theory[28].

#### The permutation entropy

Permutation entropy is proposed based on the counting of the ordinal patterns of time series. First of all, the relative frequencies of the ordinal patterns or the probability distribution of permutations is evaluated. For a time series {*x*(*t*); *t* = 1,2,⋯,*N*}, it can be sorted in an increasing order. In other words, an embedding procedure forms data segments where *n* the number of samples belonging to the segment is. Therefore, a series of vectors of length *n* is derived from {*x*(*t*)}:

$$\vec{x}(i)=[x(i),x(i+\tau ),x(i+2\tau ),\cdots ,x(i+(n-1)\tau )];\;i=1,2,\cdots ,N-(n-1)\tau$$ (5)

Then, $\vec{x}(i)$ is arranged in an increasing order of magnitudes:

x→'(i)=[x(i+j1−1),x(i+j2−1),⋯,x(i+jn−1)] (6)

Obviously, (*j*_1_,*j*_2_,⋯,*j*_*n*_) is a permutation of (1,2,⋯,*n*).

In this way, there are *n*! permutations of order *n*, which are considered as all possible order types of *n* different numbers. Accordingly, each $\vec{x}(i)$ is mapped to one out of *n*! permutations. When each permutation is considered as a symbol *π*_*j*_, the reconstructed trajectory is represented by a symbol sequence. The number of distinct symbols could be at most *n*!. For each symbol *π*_*j*_, let *f*(*π*_*j*_) denotes its frequency of occurrence in the time series. Consequently, the probabilities *p*(*π*_*j*_) of the occurrence of each symbol are obtained from the reconstructed sequences.

$$p(\pi_{j})=\frac{f(\pi_{j})}{N-(n-1)\tau };\;j=1,2,\cdots ,m,m\leq n!$$ (7)

And then, the permutation entropy of {*x*(*t*)} is defined as [23, 29]:

$$H\left(X\right)=-\sum p\left(\pi_{j}\right)\log p\left(\pi_{j}\right)$$ (8)

For two time series {*x*(*t*)} and {*y*(*t*)}, let *H*(*X*) and *H*(*Y*) denote their permutation entropies. The joint probability $p(\pi_{j}^{x},\pi_{j}^{y})$ of each symbol occurrence in the signals can be calculated [30]. Thus the joint permutation entropy *H*(*X*,*Y*) is defined as:

$$H\left(X,Y\right)=-\sum_{\pi_{j}^{x}}\sum_{\pi_{\text{k}}^{y}}p(\pi_{j}^{x},\pi_{k}^{y})\log\;p(\pi_{j}^{x},\pi_{k}^{y})$$ (9)

And later the mutual information on account of permutation entropies can be achieved as following:

I(X,Y)=H(X)+H(Y)−H(X,Y) (10)

#### Calculating PMI

The mutual information of a fast oscillation amplitude *A*_*High*_(*t*) and a slow oscillation phase *θ*_*Low*_(*t*) can be measured by the PMI method, which is developed to evaluate the PAC between two frequency bands (oscillations). However the phase series *θ*_*Low*_(*t*) of the slow oscillation *Low*(*t*) is a periodic function of time points and also monotonically increasing in each period. Therefore, the permutation patterns of *θ*_*Low*_(*t*) would be very simple, which is poor for analyzing the coupling between *θ*_*Low*_(*t*) and *A*_*High*_(*t*). On the contrary, it can be seen that the magnitude orders of cos(*θ*_*Low*_(*t*)) are similar to that of *A*_*High*_(*t*) if there is a significant PAC between them. Accordingly, PMI between cos(*θ*_*Low*_(*t*)) and *A*_*High*_(*t*) would be benefit for measuring PAC.

In fact, by using Hilbert transformation,

Low(t)=Re(ALow(t)*eiθLow(t)) (11)

*A*_*Low*_(*t*) and *θ*_*Low*_(*t*) are the amplitude and the phase series of the slow oscillation, respectively.

Thus cos(*θ*_*Low*_(*t*)) can be generated by dividing *Low*(*t*) by its instantaneous amplitude *A*_*Low*_(*t*):

Low(t)ALow(t)=Re(eiθLow(t))=cos(θLow(t)) (12)

Therefore, the magnitude order of cos(*θ*_*Low*_(*t*)) just depends on the phase series of slow oscillation, which is benefit for measuring PAC.

After producing the phase series cos(*θ*_*Low*_(*t*)), we further apply Hilbert transformation in generating the amplitude *A*_*High*_(*t*) of the fast oscillation *High*(*t*). The PMI of two time series can be measured as follows:

$$PMI=I(\cos(\theta_{Low}(t)),A_{High}(t))$$ (13)

*I*(•) denotes the mutual information of these two time series based on permutation entropy. The order *n* of permutation entropy used hereby is three.

Therefore, the value of PMI is able to quantify the modulation intensity of the slow oscillation phase to the fast oscillation amplitude. If the amplitude of high frequency oscillation is independent of the phase of slow oscillation, the PMI will be close to 0. Otherwise, if the fast oscillation amplitude envelop has the same shape as the slow oscillation, the PMI will be equal to the self-information *H*(cos(*θ*_*Low*_(*t*))) of slow oscillation phase series based on permutation entropy.

Moreover, *PMI* ≤ min{*H*(cos(*θ*_*Low*_(*t*))),*H*(*A*_*High*_(*t*))} ≤ *H*(cos(*θ*_*Low*_(*t*))), thus the PMI value can be further normalized by *H*(cos(*θ*_*Low*_(*t*))):

$$PMI_{Nom}=\frac{PMI}{H(\cos(\theta_{Low}(t)))};\;PMI_{Nom}\in [0,1]$$ (14)

Or we may simply define the PMI as follows,

$$PMI=\frac{I(\cos(\theta_{Low}(t)),A_{High}(t))}{H(\cos(\theta_{Low}(t)))};\;PMI\in [0,1]$$ (15)

On the one hand, the PMI will be zero if the two time series are independent of each other. On the other hand, the PMI value will be the largest one if these two time series come from the same permutation distribution with same permutation orders. In the present study, the PMI value is always the normalized one.

### Construction of phase-amplitude plot

The phase-amplitude plot was a by-product when applying the modulation index (MI) approach in signals to measure PAC. Specifically, when using MI, each cycle of the phase of the slow oscillation is equally divided into several intervals (n = 18 in the present study). Corresponding to each phase interval, the average of the fast oscillation amplitude can be calculated. Therefore an amplitude distribution *P* of fast oscillation conditioned on the slow oscillation phase could be derived. The phase-amplitude plot is obtained by plotting the amplitude distribution as a function of the phase interval [22].

### Acquisition of simulation data

Let *low*(*t*) and *high*(*t*) denote the slow oscillation and the fast oscillation that are used to construct raw data, compared to *Low*(*t*) and *High*(*t*) that are oscillations filtered from raw data.

Firstly, a stable slow oscillation *low*(*t*) and a stable fast oscillation *high*(*t*) are generated for each time point *t*,

$$low(t)=\sin(\frac{f_{low}}{f_{s}}*2\pi t),\;high(t)=\sin(\frac{f_{high}}{f_{s}}*2\pi t)$$ (16)

Here, the parameter *f*_*low*_ denotes the center frequency of slow oscillation, and *f*_*high*_ represents the center frequency of fast oscillation. The sampling rate is *f*_*s*_.

The Von-Mises Coupling method [31] is applied to produce the amplitude series of high frequency oscillation:

$$A_{high}(t)=\frac{c}{\exp\;\lambda }\exp[\lambda \cos(\theta_{low}(t)-\theta_{0})]$$ (17)

The parameter *c* controls the maximum amplitude of high frequency oscillation. *θ*_0_ is the phase where *A*_*high*_(*t*) reaches its maximum value, which is called modulation phase or preferred phase of PAC. *λ* is a concentration parameter. The bigger value of *λ* generates the larger amplitude of high frequency oscillation only at the phases closed to modulation phase *θ*_0_, and a zero value of *λ* causes equally the large amplitudes of fast rhythm at all phases *θ*_*low*_(*t*). In the present study, *c* = 1, *λ* = 0.95, and $\theta_{0}=\frac{\pi }{2}$ [21].

Afterwards, raw data with PAC could be generated as:

$$raw=low(t)+A_{high}(t)\;*\;high(t)+\sigma \;*\;WN$$ (18)

*WN* is the Gaussian white noise with a zero mean and a standard deviation *σ*.

**Fig 2:**
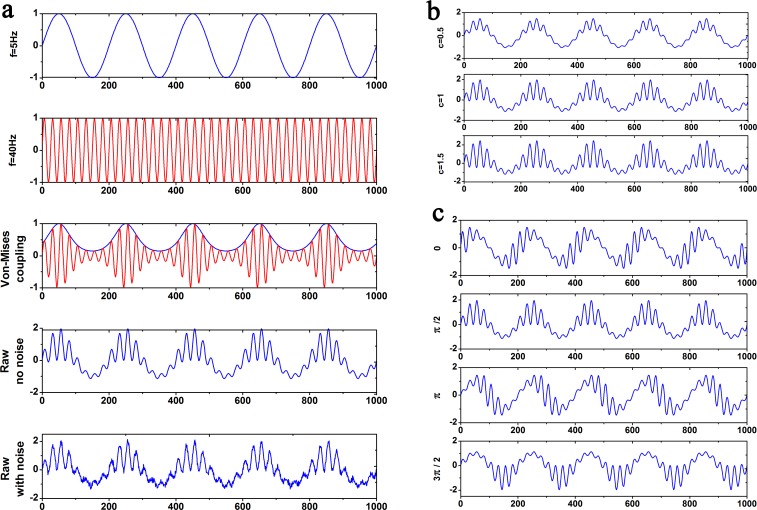
Simulation data were generated by Von-Mises Coupling. (a) From top panel to bottom panel: a 5*Hz* slow oscillation; a 40*Hz* fast oscillation; a 40 Hz fast oscillation, whose amplitude was modulated by the phase of 5*Hz* slow oscillation; Simulation data without noise; simulation data with noise (*σ* = 0.1). (b) Simulation data with different absolute amplitude levels of fast rhythm. The parameter *c* was increased from 0.5 to 1.5. (c) Simulation data with different modulation phases. The parameter *θ*_0_ was changed from 0*π* to $\frac{3}{2}\pi$.

If a particular assumption is not made, a fixed combination of frequencies is utilized throughout simulations (a 40 Hz oscillation is modulated by a 5 Hz oscillation) to model the theta-gamma PAC and the sampling frequency *f*_*s*_ is 1000 *Hz*. A detailed process for creating raw data can be seen in Fig 2A. Fig 2B shows the raw data with different amplitude of fast oscillation. They are generated by increasing the parameter *c* from 0.5 to 1.5. Moreover, Fig 2C shows the raw data with different modulation phases, which are produced by changing the parameter *θ*_0_ from 0 to $\frac{3}{2}\pi$.

Based on the three types of interference with PAC measurement, the above raw data are reconstructed with interferential signals, which are added into slow or fast oscillation. The interferential signals come from two ways: a sinusoidal oscillation or a filtered oscillation of experimental data. The interferential sinusoidal oscillation is a 7 Hz oscillation for slow rhythm and a 44 Hz oscillation for fast rhythm. In addition, the interferential oscillation from experimental LFP data is a filtered 4~6 Hz theta oscillation or a filtered 38–42 Hz gamma oscillation, either of which is normalized to a range from -1 to 1. The reconstruction procedures of raw data are described separately in result sections in the paper.

### Data analysis

After generating the reconstructed raw data, they are filtered into two frequency bands to generate *Low*(*t*) and *High*(*t*) used to measure the performance of PAC methods. All filters in the study are realized by means of eegfilt.m from EEGLAB toolbox [32]. The center frequency of slow oscillations is 5 Hz and the filter bandwidth is 6 Hz. The center frequency of fast oscillations is 40 Hz and the filter bandwidth is 12 Hz. A significant PAC could be achieved if the bandwidth of high frequency is at least twice the center frequency of low frequency oscillation [33].

Subsequently, the filtered oscillations obtained from simulation data are analyzed by the three approaches while changing the proportion of interferential oscillation. The length of data is fixed as 10 s and 20 trials are run for each set of parameters under study (coupling intensity k and noise level *σ*). The PAC intensity k means the proportion of slow or fast oscillation that is actually involved in the concerned PAC phenomenon. PAC is measured when changing the PAC intensity k from 0 to 1 by an increase of 0.1. For each method, the PAC values are normalized by the average of PAC values when k = 1.

### Acquisition of experimental data

Experimental data in Fig 1 was obtained from an adult C57 mouse(S1 Mat). The animal did not become ill prior to the experimental endpoint. The mouse was administered with urethane anaesthesia (Sigma-Aldrich, St. Louis, MO, USA; 1.2 g/kg body weight; supplemental doses of 0.2–0.8 g/kg as needed). It was positioned in a stereotaxic apparatus (Narishige, Japan) with a heating pad for surgery. A mouse brain atlas [34] was used for the stereotaxic coordinates of electrodes. Small holes in the skull were drilled. A monopolar stainless steel recording electrode was positioned in the granule cell layer of the DG (1 mm lateral and 1.7 mm posterior to the bregma, 1.8 mm from the brain surface). The local field potentials (LFPs) were recorded at the hippocampal DG region with a sampling rate of 1000 Hz for one hour. All procedures were performed in accordance with the Ethical Commission at Nankai University, China.

Experimental data were also obtained from two male Wistar rats (S2 Mat). One was in adolescence (180g) and another was in adulthood (360g). The animals did not become ill prior to the experimental endpoint. The experiment was performed at the College of Life Sciences, Nankai University, P. R. China. The animal was anesthetized by 30% urethane (4ml/kg, i.p., Sigma-Aldrich, St.Louis, MO, USA) and then was placed in a stereotaxic frame (Narishige,Japan). A small hole (2 mm in diameter) was made on its left hemisphere. Two stainless electrodes were planted into the hippocampal CA3 (4.2 mm posterior to the bregma, 3.5 mm lateral to midline, 2.5 mm ventral below the dura) and CA1 (3.5 mm posterior to the bregma, 2.5 mm lateral to midline, and 2.0 mm ventral below the dura) areas. Ground and reference electrodes were placed symmetrically over the two brain hemispheres. The local field potentials were collected at both hippocampal CA3 and CA1 regions with a sampling frequency of 1000 Hz for 1 hour. All procedures were carried out in accordance with the Ethical Commission at Nankai University, China.

We would like to emphasize that all animals were anesthetized, by which they could be maintained in stable state about 6–10 h. Meanwhile, a electric warm blanket was used to maintain the animals’ body temperature. In addition, we usually checked animal’s chest abdominal breathing for every 10–15 min during the experiment. Furthermore, Every effort has been made to minimize the number of animals used and their suffering, which included the use of proper anesthesia. For example, we frequently assess both the corneal and tail withdrawal reflex of animals. Only under the condition of the absence of both corneal and tail withdrawal reflex, which usually happens about 30 mins after urethane administration, anesthetized animals were placed in a stereotaxic frame. This process has been done in order to minimize their suffering. After the electrophysiological experiments, the animals were sacrificed by cervical dislocation.

In the present study, the interferential 4~6 Hz theta oscillations were filtered from CA1 LFP of the puberty rat and CA3 LFP of the other adult rat because these two LFP had a power peak in 4~6 Hz frequency range. The interferential 38~42 Hz gamma oscillations were filtered from the same source but there was not a power peak in 38~42 Hz range. 40 epochs were obtained randomly from the filtered signal (20 epochs from CA1 LFP and 20 epochs from CA3 LFP). The length of each epoch was 10 s and there was not an overlap between any two epochs.

## Results

### Examples of interferential oscillations from an adult mouse

The MI approach was employed to analyze PAC of the experimental LFP data, obtained from the hippocampal DG region in a mouse (Fig 1). Data were showed by PAC comodulograms. They were constructed by both *f*_*low*_ and *f*_*high*_, where *f*_*low*_ was firstly generated from 1 Hz to 10 Hz in 1 Hz step with 1 Hz bandwidth and *f*_*high*_ from 30 Hz to 80 Hz in 1 Hz step with 1 Hz bandwidth (Fig 1A). Moreover, the comodulograms were also constructed by filtering *f*_*low*_ with 2 Hz bandwidth (Fig 1B) or filtering *f*_*high*_ with 2 Hz bandwidth (Fig 1D). Therefore, there were two pairs of PAC comoduligrams, i.e. Fig 1A & 1B and Fig 1A & 1D. In order to show more clearly the interference with PAC measurement, subtractions were done between each pair of comodulograms (Fig 1C and 1E).

It can be seen that there are three types of PAC interference. They are: (i) the slow oscillation band covers two different components, one of which modulates the fast oscillation amplitude and another not. In Fig 1A, a dominant PAC exists between 3~5Hz theta phase and gamma (30~45 Hz and 55~80 Hz) amplitude. There is no PAC phenomenon between 2~3 Hz theta and gamma. When the slow oscillation is filtered with 2 Hz bandwidth (Fig 1B), there are obvious decreases of MI values indicating the PAC intensities between 3~4Hz theta and gamma (Fig 1C). The decreases of MI values perhaps result from the interference of 2~3 Hz oscillation, which is irrelevant to PAC phenomenon (Fig 1A). (ii) the fast oscillation band covers two different components, one of which is modulated by the slow oscillation phase and another not. If the gamma frequency band is filtered with a 2 Hz bandwidth (Fig 1D), the PAC measurement is feasibly interfered with the presence of possible interferential gamma at nearby frequency (Fig 1E). (iii) the fast oscillation band only consists of one component, whose amplitude, however, is jointly modulated by two slow oscillation phases. In Fig 1A, it can be seen that 40 Hz gamma amplitude is modulated by both 3~4 Hz and 4~5 Hz theta rhythms, respectively. When the PAC intensity between 3~4 Hz theta and 40 Hz gamma is assessed by the PAC approaches, the PAC measurement could be disturbed by the 4~5 Hz theta.

Although the above examples of interferential oscillations occur at different frequency bands, they may have the same frequency band as PAC-involved oscillations in experimental data, and are hard to be illustrated in the PAC comodulograms.

### Application to simulation data

Clearly, the annotation of PAC intensity needs to be verified. Therefore, the proposed intensity was measured by the three approaches in order to detect a positive correlation between the theoretical intensity and the calculated PAC values. Since there are three types of interferences with PAC measurement, simulation data are generated in three different ways, accordingly. For each type of simulation, the interference component of slow or fast oscillation comes from two ways, which are a sinusoidal oscillation and a filtered oscillation from experimental data.

#### Simulation type I: A slow oscillation band consists of two different components, only one of which modulates a fast oscillation amplitude

The reconstructed raw data are generated as follows,

$$raw=k\;*\;low_{5Hz}(t)+(1-k)low_{7Hz}(t)+A_{high}(t)\;*\;high_{40Hz}(t)+\sigma \;*\;WN$$ (19)

The slow rhythm consists of two oscillation components, a 5 *Hz* sinusoidal oscillation *low*_5 *Hz*_(*t*) and a 7 *Hz* sinusoidal oscillation *low*_7 *Hz*_(*t*), respectively. *A*_*high*_(*t*) is produced by Von-Mises Coupling, which is merely modulated by the phase of *low*_5 *Hz*_(*t*). The parameter *k* ∈ [0,1] is a coefficient representing the ratio of *low*_5 *Hz*_(*t*) to the slow rhythm, which is regarded as the intensity of PAC. Obviously, *low*_7 *Hz*_(*t*) is a factor interfering with PAC measurement. Accordingly, the raw data were generated by changing the PAC intensity parameter k, which varied from 0 to 1 by an increase of 0.1.

**Fig 3:**
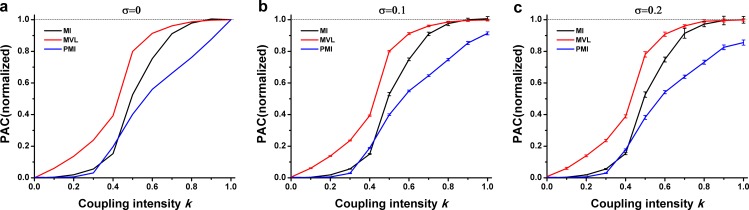
Performance of three PAC approaches in simulation type I. **The interferential oscillations were sinusoidal oscillations.** (a) Simulation data were constructed without noise. The PAC intensity *k* varied from 0 to 1. Each curve was averaged over 20 trails. PAC values derived from three approaches were normalized by the average of PAC values when *k* = 1. This simulation procedure was utilized throughout the present study. (b) Noise level *σ* = 0.1. (c) Noise level *σ* = 0.2.

Fig 3 showed that there was a positive relationship between the PAC values and the intensity parameter k. It could be seen that the MVL was nearly proportional to k when k ≤ 0.4, and considerably increased when k was changed from 0.4 to 0.7. When k ≥ 0.8, it was maintained in high values. For both the MI and the PMI, the value of PAC measurements is not correspond to the PAC intensity when k ≤ 0.2. While k was changed from 0.3 to 0.8, the MI values were greatly increased to nearly 1. In the same way, it was maintained in high values after k ≥ 0.8. However, the value of PMI measurement was linearly increased after k ≥ 0.3. The comparable results were obtained under the different levels of noise (Fig 3B & 3C). It should be noted that the PMI was sensitive to noise when k ≥ 0.9.

The reconstructed raw data could also be generated as follows,

$$raw=k\;*\;low_{5Hz}(t)+(1-k)low_{4\sim 6Hz}(t)+A_{high}(t)\;*\;high_{40Hz}(t)$$ (20)

The slow rhythm consists of two oscillation components, which are a 5 *Hz* sinusoidal oscillation *low*_5 *Hz*_(*t*) and a 4∼6 *Hz* oscillation *low*_4∼6 *Hz*_(*t*) filtered from experimental LFP. *A*_*high*_(*t*) is only modulated by the phase of 5 Hz slow oscillation *low*_5 *Hz*_(*t*). The parameter *k* is the intensity of PAC.

**Fig 4:**
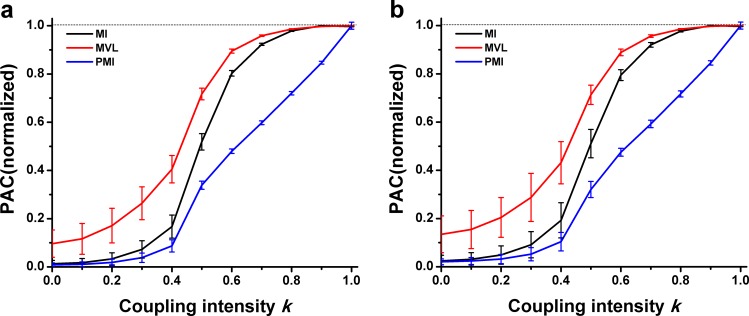
Performance of three PAC approaches in simulation type I. **The interferential oscillations were filtered from experimental LFP.** (a) The interferential oscillations were filtered from the CA1 LFP of the puberty rat. (b) The interferential oscillations were filtered from the CA3 LFP of the adult rat.

Fig 4 showed that there was a positive relationship between the PAC values and the intensity k when the interferential oscillations were filtered from experimental LFP. No matter whether the interferential oscillations were come from the signals of the CA1 LFP of a puberty rat (Fig 4A) or from the CA3 LFP of an adult rat (Fig 4B), the performances of three approaches were nearly similar to that when the interferential oscillations were sinusoidal oscillations (Fig 3). It was found that, however, the PAC intensity could not be correctly measured by the MVL while k ≤ 0.3.

As illustrated in both Fig 3 and Fig 4, the proposed annotation of PAC intensity worked well when the interfering oscillation was a component of slow oscillations.

#### Simulation type II: A fast oscillation band consists of two different components, only one of them is modulated by a slow oscillation phase

The reconstructed raw data were generated as following,

$$raw=low_{5Hz}(t)+k\;*\;A_{high}(t)\;*\;high_{40Hz}(t)+(1-k)\;*\;high_{44Hz}(t)+\sigma \;*\;WN$$ (21)

The fast rhythm consists of two oscillation components, a 40 *Hz* sinusoidal oscillation *high*_40*Hz*_(*t*) and a 44 *Hz* sinusoidal oscillation *high*_44*Hz*_(*t*). The amplitude *A*_*high*_(*t*) of the 40 *Hz* oscillation is only modulated by the phase of the 5 *Hz* slow oscillation. The parameter *k* ∈ [0,1] is a coefficient representing the ratio of *high*_40*Hz*_(*t*) to the fast rhythm, which is regarded as the intensity of PAC. The 44 *Hz* fast oscillation is a factor interfering with PAC measurement.

**Fig 5:**
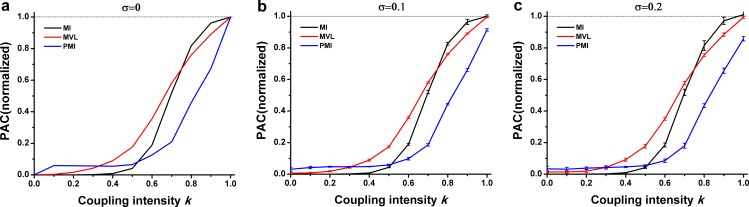
Performance of three PAC approaches in simulation type II. **The interferential oscillations were sinusoidal oscillations.** (a) Simulation data were constructed without noise. (b) Noise level *σ* = 0.1. (c) Noise level *σ* = 0.2.

Fig 5 showed that all the three methods could not properly detect PAC when the coupling intensity was relatively weak. Among them, the MVL performed comparatively well. Particularly, the PAC intensity could be properly detected by the MVL approach after k ≥ 0.3. It can be seen that it almost linearly increases when k changes from 0.4 to 1. Furthermore, the MI value was close to 0 while k ≤ 0.4, and the PMI value was also small when k ≤ 0.5, though a large value could be obtained by the MI and PMI approaches when the coupling intensity was strong.

The reconstructed raw data could also be generated as following,

$$raw=low_{5Hz}(t)+k\;*\;A_{high}(t)\;*\;high_{40Hz}(t)+(1-k)\;*\;high_{38\sim 42Hz}(t)$$ (22)

The fast rhythm consists of two oscillation components, a 40 *Hz* sinusoidal oscillation *high*_40 *Hz*_(*t*) and a 38 ∼ 42 *Hz* oscillation *high*_38∼42 *Hz*_(*t*), which are obtained from experimental LFP signals. It supposes that only the 40 *Hz* fast oscillation amplitude is modulated by the phase of 5 Hz slow oscillation. The parameter k represents the intensity of PAC, too.

**Fig 6:**
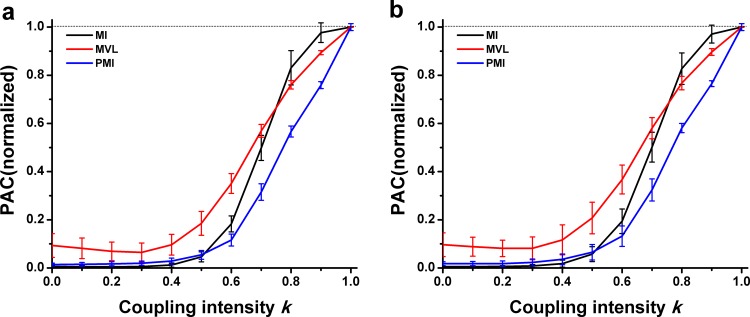
Performance of three PAC approaches in simulation type II. **The interferential oscillations were filtered from experimental LFP.** (a) The interferential oscillations were filtered from the CA1 LFP of the puberty rat. (b) The interferential oscillations were filtered from the CA3 LFP of the adult rat.

Fig 6 shows that there are different performances of these three approaches when the interferential oscillations are come from the experimental LFP. It was found that the PAC was not properly measured by the MVL approach when k ≤ 0.4. However, the PAC was able to be correctly measured by the three approaches after k ≥ 0.5. Moreover, the performances of both the MI and PMI were in line with their performance when the interferential oscillations were sinusoidal oscillations.

In spite of the fact that there is not an exact positive correlation between the PAC values and the proposed PAC intensity (Fig 5 and Fig 6), which may be due to the large signal noise ratio (SNR) when the interfering oscillation is a high frequency oscillation, the truth is that the proposed annotation reveals the PAC intensity to some extent.

#### Simulation type III: a fast oscillation band consists of one component, whose amplitude, however, is jointly modulated by two slow oscillations

The reconstructed raw data were generated as following,

$$raw=low_{5Hz}(t)+k\;*\;A_{high}^{5Hz}(t)\;*\;high_{40Hz}(t)+(1-k)\;*\;A_{high}^{7Hz}(t)*high_{40Hz}(t)$$ (23)

The fast rhythm consists of one oscillation component, which is a 40 *Hz* sinusoidal oscillation *high*_40*Hz*_(*t*). In addition, the amplitude of the 40 *Hz* oscillation is jointly modulated by the phases of both a 5 *Hz* sinusoidal oscillation and a 7 *Hz* sinusoidal oscillation. The parameter *k* represents the PAC intensity. An impact that 40 Hz oscillation amplitude is modulated by 7 *Hz* oscillation phase certainly interferes with the PAC measurement between 5 Hz oscillation phase and 40 Hz oscillation amplitude.

**Fig 7:**
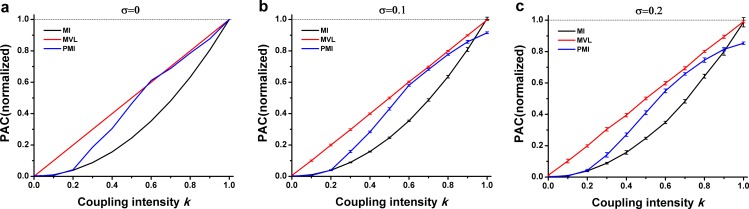
Performance of three PAC approaches in simulation type III. **The interferential oscillations were sinusoidal oscillations.** (a) Simulation data were constructed without noise. (b) Noise level *σ* = 0.1. (c) Noise level *σ* = 0.2.

Fig 7 showed an ideal performance that the MVL measurement was found to be proportional to k. Visibly, there was a parabolic trend for the MI measurements. Moreover, the PMI measurements were more or less between them.

The reconstructed raw data could also be generated as following,

$$raw=low_{5Hz}(t)+k\;*\;A_{high}^{5Hz}(t)\;*\;high_{40Hz}(t)+(1-k)\;*\;A_{high}^{4\sim 6Hz}(t)*high_{40Hz}(t)$$ (24)

The fast rhythm consists of one oscillation component, which is a 40 *Hz* sinusoidal oscillation *high*_40*Hz*_(*t*). In addition, the amplitude of the 40 *Hz* oscillation is jointly modulated by the phases of both a 5 *Hz* sinusoidal oscillation and a 4∼6 *Hz* oscillation, obtained from experimental LFP signals. The 4∼6 *Hz* oscillation phase modulation for 40 Hz fast oscillation was a factor interfering with PAC measurement between the 5 Hz oscillation phase and the 40 Hz oscillation amplitude.

**Fig 8:**
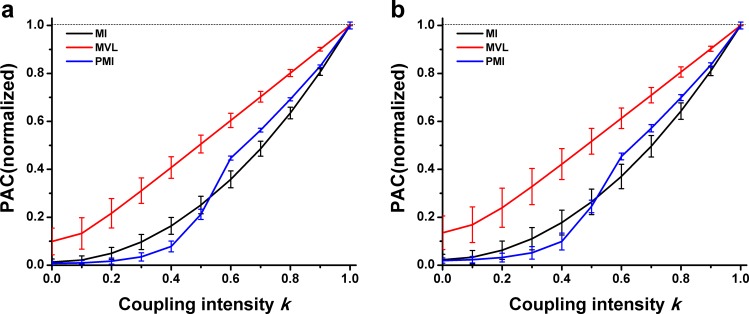
Performance of three PAC approaches in simulation type III. **The interferential oscillations were filtered from experimental LFP.** (a) The interferential oscillations were filtered from the CA1 LFP of the puberty rat. (b) The interferential oscillations were filtered from the CA3 LFP of the adult rat.

Fig 8 showed the relationship between the value of PAC measurements and the coupling intensity *k* when the interferential oscillations were obtained from experimental LFP signals. The result was similar to that of the previous situation, where the interferential oscillations were sinusoidal oscillations. However, the PAC measurements could not be performed properly by the MVL approach when *k* ≤ 0.3.

Obviously, the PAC values derived from the three methods were proportional to the proposed intensity (Fig 7 and Fig 8). Among all these three methods, the MVL measurement showed the best performance that there was an absolutely positive relationship between MVL values and the proposed intensity. The results suggest that the proportion of oscillations actually involved in PAC is able to represent the PAC intensity between the concerned frequency bands.

### Phase-amplitude plots

Although the three types of simulations show that the annotation of PAC intensity is proper to some extent, it is necessary to make sure that the annotation does not conflict with the widely accepted understanding of PAC intensity, i.e. the uneven distribution of fast oscillation amplitude [7, 11, 12, 35]. Accordingly, the phase-amplitude plots were constructed as another exemplification that the annotation could denote the PAC intensity.

**Fig 9:**
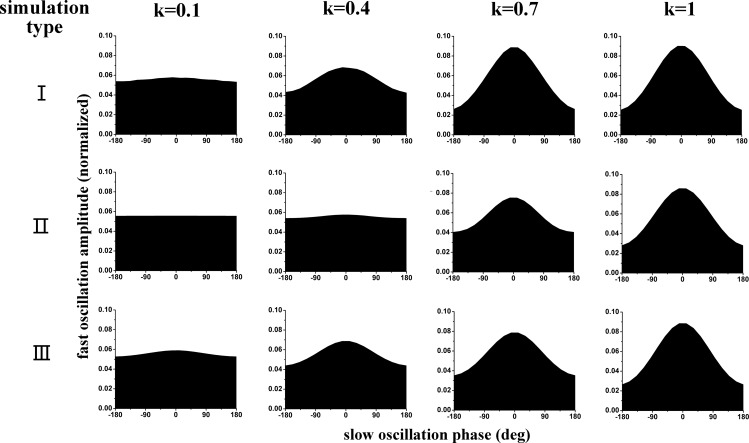
The phase-amplitude plots for the three simulation types and different coupling intensity k.

Fig 9 showed the phase-amplitude plots for the three simulation types and different coupling intensity *k*, which was ranged from 0.1 to 1. It could be seen that the distribution pattern of fast oscillation amplitude tended to be a more uneven along with the increase of PAC intensity *k*. According to our understanding, the PAC intensity can be inferred by visual inspection of the phase-amplitude plot. Generally speaking, the more uneven distribution the stronger coupling. The results implied that the annotation of PAC intensity, proposed in the study, was suitable while the parameter *k* was able to characterize the intensity of PAC.

## Discussion

The PAC intensity was measured by a number of approaches, however, there was not a clear conception of it. The aims of the study was to give a precise annotation of the PAC intensity for much accurate estimation of PAC intensity. A numerical test shows that the similar performance has been achieved by the MI, MVL or PMI approaches when measuring PAC intensity. The values of PAC intensity, obtained from these three PAC approaches, are positively proportional to the proposed intensity. In addition, the phase-amplitude plots show that the annotation is in line with previous understanding of PAC intensity.

More and more experimental evidences show that the phase synchronization and cross-frequency coupling (CFC) are able to support neural communication and facilitate neural plasticity [7]. Cross frequency phase-amplitude coupling, which is also referred to oscillatory synchronization phenomenon, has been widely reported in the last decade [4, 9, 10] and is relative to both multi-item working memory maintenance and long-term memory retrieval [11, 12]. For example, the theta-gamma PAC is regarded as the main mechanism for the organization of serial memories and maintenance of working memories [19].

Several kinds of approaches have been employed to measure the intensity of PAC. Each method has been developed based on the respective theory and for the distinctive purpose. For example, the approach of cross-frequency phase locking value (CF-PLV) was proposed by the idea “phase synchronization” between a slow oscillation and the amplitude envelop series of a fast oscillation [21, 36]. In addition, the method of general linear model (GLM) [21] was to linear fit the amplitude of high frequency oscillation at the phase of low frequency oscillation. The significances of PAC intensity in various PAC methods are different from each other. It is generally understood that the PAC intensity is mainly related to the uneven distribution of the fast oscillation amplitude. The more uneven distribution usually represents the higher PAC intensity. It is well known that both the approaches of MVL and MI are developed based on such an idea [11, 14, 35]. Moreover, the normalized PMI method was developed to evaluate the PAC intensity in this study, by which the information rate of communication channel between slow oscillation phases and fast oscillation amplitudes was used to denote PAC intensity.

As illustrated in Fig 2, there are three types of interferences that may significantly impact PAC measurements with regard to real experimental data. These interferences can not be avoided because it is hard to distinguish signals, derived from different sources but in the same frequency band, by commonly used filtering techniques. In addition, for EEG or LFP signals, slow oscillation modulates activity over large spatial regions in long temporal windows, whereas fast oscillation modulates activity over small spatial regions and short temporal windows [37]. As a result, there would be inevitable interferences with PAC phenomenon. In the present study, the proportion of signals that are actually involved in PAC phenomenon is regarded as theoretical PAC intensity. Thus, the existence of the interferential oscillation component will have a negative impact on PAC intensity. Weak or strong coupling between two frequency bands could be easily derived from different proportions mentioned above. It should be noted that the annotation of PAC intensity in the study was made only in view of our limited understandings of PAC.

Furthermore, the simulation study was performed, in which there were three types of PAC with interference. In the type I of simulation study, it was assumed that the slow oscillation consisted of two components, one of which modulated the amplitude of fast oscillation. Indeed, it was found that there were two independent theta oscillation components in hippocampus [38]. One was generated in subiculum and the other in the CA3 region. Both of them flowed throughout the hippocampus. For PAC measurement, its intensity between one theta component and a fast oscillation (e.g. gamma oscillation) would be suppressed by the other theta. In the simulation type II, the fast oscillation consisted of two components. Just like the simulation type I, only one component of the fast oscillation was modulated by the slow oscillation phase. In the high frequency band of EEG or LFP signals, the power frequency noises may interfere with the functional oscillation bands of PAC, which could be removed by a point-filter. However, there were also pink noises that would be possible sources for interferential oscillations. Besides, a fast oscillation band may be divided into several sub bands since their different functions and different generation sources. For example, gamma oscillations in the hippocampus are usually separated into three bands, i.e. the slow gamma(30–80 Hz), the mid-frequency gamma (60–120 Hz) and the fast gamma (>100 Hz) [39]. It could be seen that there were overlaps between the sub bands of gamma. So the PAC intensity between theta and one sub band of gamma would be interfered by other sub band of gamma during the PAC measuring. Perhaps the most clear interference type would be the simulation type III because it could be derived from the PAC comodulograms directly. In the most of PAC studies[4, 9, 14, 15, 35, 40], the frequency range of slow oscillations band that significantly modulated fast oscillation was not just a single frequency (bandwidth< 1 Hz). Therefore, the fast oscillation may be modulated by two slow oscillations, respectively. For example, if the gamma amplitude is modulated by theta oscillations at different frequencies, the PAC measurement between gamma and one of theta oscillations may be interfered with the presence of the other theta oscillation. It should be noted that even if the slow oscillations are filtered without the interference of the simulation type I, the fast oscillation would still be modulated by two slow oscillations, which may lead to a reduction of PAC intensity. In a word, the interferences with PAC are common in neural oscillations, hence, the novel annotation of the PAC intensity has been well-founded to some extent.

Simulation data generated with different PAC intensities are analyzed by three PAC approaches. The three measures are estimated in different domains: MVL works with real numbers in the continuous domain, MI is based on discretization of the phase into several bins, while PMI is based on permutation approach using patterns of increase/decrease of a few subsequent samples. The results show that there is a positive correlation between PAC intensity *k* and PAC values calculated by the PAC approaches (Figs 3–8). It is well known that both the MVL and MI approaches have widely been employed to investigate PAC phenomenon. Moreover, the mutual information has also been applied in measuring correlation or coupling. Thus, it is credible to make such a novel annotation for the PAC intensity. To make sure that the annotation is consistent with the common understanding of PAC intensity, the phase-amplitude plots are utilized for clarifying different intensities. In each simulation type, a higher intensity always corresponds to a more uneven distribution of fast oscillation amplitude (Fig 9). Since the phase-amplitude plot has widely been used to denote PAC intensity, the annotation of PAC intensity proposed in the present study is proper in this view to some extent.

It should be noted that the three types of interferences may be concurrent in experimental situations. Whether the proposed annotation of PAC intensity is suitable in these situations is still unclear. Moreover, as the PAC values derived from some kind of approaches are possibly interfered by the irrelevant oscillations (Fig 1), the commonly used PAC comodulograms may not reflect PAC intensity correctly. Although the aim of the present study was not to explore a better way for using PAC methods, it was also worth pointing out that the different performances of MVL (when the sources of interferential oscillation were different) indicated that simulations of PAC, if only derived from sinusoid oscillations, perhaps could not represent the actual experimental PAC. It was suggested to utilize experimental data to construct simulation data.

## Conclusion

In this study, we proposed a novel annotation of the PAC intensity associated with the interferential oscillations. A numerical test based on simulation data shows that the similar performance has been achieved by these three PAC approaches when measuring PAC intensity. The values of PAC intensity, obtained from these three PAC approaches, are positively proportional to the theoretical intensity, suggesting that the annotation is proper to some extent. Finally, the phase-amplitude plots show that the annotation is in line with previous understanding of PAC intensity.

## Supporting Information

S1 Mat LFP data recorded in PP and DG regions from a mouse. (MAT)

S2 Mat LFP data recorded in CA3 and CA1 regions from two rats. (MAT)
